# London Hospital Problems

**Published:** 1907-07-20

**Authors:** 


### HOSPITAL ADMINISTRATION.

### CONSTRUCTION AND ECONOMICS.

### LONDON HOSPITAL PROBLEMS.

### WHAT CONSTITUTES THE ESSENTIALS OF A MODERN GENERAL HOSPITAL?

There would seem to be many members of the British Medical Association resident in Hampstead who do not possess a clear conception of the essentials of a modern general hospital. We arrive at this conclusion from the terms of a resolution passed by the Hampstead branch of the British Medical Association on July 3, which insists that the Hampstead General Hospital to contain 111 beds should always have a staff of local practitioners. The meeting, in effect, further called upon the members of the medical profession to refuse to accept any office on the staff of the Hampstead General Hospital unless the terms offered to them are considered satisfactory by the local practitioners resident in Hampstead. If these two requirements are to be treated without prejudice and entirely on their merits, they amount in effect to a declaration on the part of the local practitioners who attended the meeting on July 3 that they will not permit the Hampstead General Hospital to become an up-to-date, fully equipped general modern hospital.

A Brief History of Facts.

What is now known as the Hampstead General Hospital began in a humble way at the instance of the late Dr. Heath Strange in 1882. For eleven years it was conducted as a pay hospital or nursing home, to which the local profession sent their patients for treatment and operation. There was no out-patient department of any kind until about ten years ago, and at the end of the last century a movement was started to remove the institution to a new site, and to build thereon a modern hospital. In 1905 the new hospital buildings were completed and contained sixty-four beds. Subsequently, as we have often pointed out, it was determined to cover practically the whole site by erecting further buildings which would contain accommodation for 111 beds, including cots. The scheme provides that of this total sixty-nine beds are to be free, twenty-two are pay, and twenty are to be cots. It must be manifest to the meanest intelligence that, if any regard is to be had to the question of hospital abuse, and if the Hampstead General Hospital with 111 beds available is to fulfil any useful purpose, many or most of its patients must be other than local cases. This conclusion rests upon the fact that Hampstead down to 1905 was able to do without a modern general hospital. Another point, and it is a very important one, is that the income of the Hampstead General Hospital has never been sufficient to maintain the sixty-four beds first opened in the new buildings, and that to adequately pay its way the hospital needed an increased revenue of upwards of £4,000 a year. For the maintenance of the 111 beds in the completed buildings, £5,000 a year additional revenue will be required. If the people of Hampstead, and the local members of the profession there, were prepared to supply this revenue, and to confine the admission of patients to local cases, who, being fit objects of charity, will amply fill the whole of the wards, there might be some reasonable grounds for the maintenance of the attitude taken up by the members of the Hampstead branch of the British Medical Association on the 3rd inst. We have been unable to discover any evidence in support of either of these views. In fact, the people of Hampstead are not likely to be willing, if they are even able, to supply the whole of the revenue needed each year to maintain 111 beds in the new Hampstead General Hospital, nor is it reasonable to suppose that there are a sufficient number of really urgent and deserving cases in Hampstead itself to keep 111 beds constantly occupied throughout each year. Indeed, if this should prove to be the case, it would probably be impossible for the Council of King Edward's Hospital Fund to make large grants of money each year for the support of a purely local hospital at Hampstead.

Important Amalgamation Proposals.

Another remarkable feature in this business is the circumstance that King Edward's Hospital Fund have very wisely determined to endeavour to bring about an amalgamation between the North-West London Hospital and the Hampstead General Hospital as a matter of business and of public policy, after a full consideration of all the circumstances of both these institutions. The scheme briefly stated amounts to this. That the out-patient department shall be conducted on a site adjacent to the present North-West London Hospital buildings, and that the hospital at Hampstead shall be utilised for the reception of in-patients from the district previously served by the former institution. The business aspects of this scheme are, that such an arrangement would immediately put the Hampstead General Hospital in funds, as the North-West London Hospital would hand over to that Committee investments and cash in the bank to the amount of £5,500, with stock in trade, including surgical instruments, valued at £400 more. They would further reap the advantage of an additional revenue of upwards of £700 from annual subscriptions, and of £1,000 from donations. King Edward's Hospital Fund will make the amalgamation effective, if the conditions laid down are complied with, by contributing for five years an annual sum of £1,500, by guaranteeing the rent of the premises of the North-West London Hospital in Kentish Town Road for ten years, and by defraying the cost of adapting these premises as an out-patients' department to the extent of £2,000. To state these facts is to record the wisdom of the scheme of amalgamation now under the consideration of the governors of the Hampstead General Hospital. It is the more remarkable, therefore, that a local meeting of the medical profession held on May 24 would have rejected them but for the casting-vote of the chairman.

### Facts Which Must be Faced.

As responsible guardians of the funds entrusted to them by the public, the Council of King Edward's Hospital Fund have made it a condition of their co-operation that the staff of the new Hampstead General Hospital shall be properly constituted. A modern general hospital must be so equipped with material and men as to be capable of undertaking the immediate and adequate treatment of every surgical and medical case which may enter its wards. The medical profession is divided into general practitioners, practising physicians and practising surgeons, and specialists who devote their whole attention to some one branch of medical practice. We have never yet known an instance where a general practitioner has come forward and claimed that he is prepared, and in every way equipped, to perform every kind of surgical operation with equal facility to that possessed by the pure surgeon who devotes the whole of his time and attention to this branch of practice. In the same way, and to an almost equal extent, the relative qualifications of general practitioners, pure surgeons, pure physicians, and pure specialists for an appointment on the honorary medical staff of a modern general hospital might be illustrated and insisted upon. It seems to us that, before the local practitioners in Hampstead can maintain their right to hold office as the active and sole members of the honorary medical staff of the Hampstead General Hospital, to the exclusion of all other members of the medical profession, they must first establish their claim to be regarded as differing from the average local practitioner by the possession of the time, the experience, the knowledge and the skill of those members of the profession who devote the whole of their time and energies to pure surgery, pure medicine, and those special departments of the profession which have to be represented on the honorary staff of a modern general hospital. It is well to add that the appointment of some great and eminent members of the profession as consulting physicians and surgeons, having regard to the fact that the holders of such offices in connection with great hospitals are practically never consulted, will in no way fulfil the requirements of a new hospital, where the whole of the medical staff consists of none but local practitioners engaged in the arduous and absorbing duties entailed by general practice. If the Hampstead General Hospital is to become anything but a mere local institution, suffering from impecuniosity and dealing only with local cases, it is essential that it shall possess an honorary medical staff of the best men with the highest qualifications and experience which the wisdom of its governors can attract to the work which it is their responsible duty to adequately provide for.

